# Age at Menarche, Growth Velocity, and Adiposity Indices in Italian Girls Aged 10 to 14

**DOI:** 10.3390/children9121928

**Published:** 2022-12-08

**Authors:** Emanuela Gualdi-Russo, Natascia Rinaldo, Gianni Mazzoni, Simona Mandini, Sabrina Masotti, Stefania Toselli, Luciana Zaccagni

**Affiliations:** 1 Department of Neuroscience and Rehabilitation, Faculty of Medicine, Pharmacy and Prevention, University of Ferrara, Corso Ercole I d’Este 32, 44121 Ferrara, Italy; 2 Center for Exercise Science and Sports, University of Ferrara, 44123 Ferrara, Italy; 3 Department for Life Quality Studies, University of Bologna, 47921 Rimini, Italy

**Keywords:** adolescence, menarche, secular trend, obesity, anthropometry

## Abstract

Age at menarche (AAM) is an effective marker of puberty timing but its onset could be influenced by several intrinsic and extrinsic factors. This study aimed to assess the AAM in a sample of Italian adolescents and to investigate its association with anthropometric variables. Considering the rise in overweight/obesity worldwide, special attention was paid to a possible decrease in AAM as adiposity indices increase. A longitudinal study was carried out on 117 middle school girls in Northern Italy. Data concerning menarche and anthropometric traits (standing and sitting height, weight, waist circumference, and skinfold thicknesses) were directly collected. Lower limb length and indices of adiposity and growth were calculated. The median AAM was 11.66 (95% IC: 11.31–11.68). Age-adjusted ANCOVA between mature and non-mature girls showed significant differences in growth-related traits and WHtR. No preponderance of overweight/obesity among mature participants was found. AAM was not significantly associated with weight or the growth velocity of adiposity indices in a subsample of maturers. Moreover, the median AAM of our sample was similar to that found in women born about 60 years ago in the same region. In conclusion, in addition to a stabilization of AAM since the 1960s, our results suggest that there is no significant correlation between increased adiposity and early AAM.

## 1. Introduction

Puberty is defined as the time at which children reach secondary sexual features and reproductive capability—fully achieved in women, according to Tanner [1], only after about 12–18 months after the onset of menarche. Age at menarche (AAM), which corresponds to the date of first menstruation, is a late puberty marker in females and it is therefore an important indicator of pubertal timing. Scientific interest in the timing and tempo of puberty, and AAM specifically, is motivated by the public health implications that may arise from any changes in the maturation process.

Humans, under the same environmental conditions, are characterized by a physiological variability of 4–5 years in the age of onset of puberty in connection with genetic factors [2]. In particular, mean AAM differs among populations depending on genetics, socioeconomic and nutritional status, geographic setting, other environmental conditions, and secular trends [3,4,5]. A declining AAM has been detected in several populations of Western countries [2,6]. This pattern has also been confirmed in Italy [7], and an increase in early puberty was also recently observed with environmental factors such as the SARS-CoV-2 pandemic [8]. However, the decrease in AAM is not a new phenomenon as shown by numerous previous studies on the secular trend [9]. Tanner reported a decrease of 0.3 years per 10 years in AAM over 130 years in Europe [1]. In Italy, a retrospective study on secular changes in AAM found that the mean age at menarche of women born in 1850–1859 was 13.7 ± 2.1 years and in women born in 1950–1959 was 11.9 ± 1.4 years. During this 100-year period, the trend has shown an acceleration in the last few decades [10]. 

The age of sexual maturation seems to have stabilized in the majority of developed countries since at least the 1990s [2,7]. However, taking into account environmental influences (particularly overeating and a sedentary lifestyle), a negative relationship between adiposity indices and the timing of menarche was found: AAM decreases as adiposity increases [11]. Thus, given the close relationships between nutritional status and the reproductive system, an important issue requiring further investigation is whether the ongoing epidemic of childhood obesity [12,13] will persist in population trends toward the earlier onset of female puberty. Indeed, there are adverse public health implications of early menarche. In general, the timing of pubertal development in girls is believed to have a deep influence on their overall health and, in particular, on their risk of long-term cardiometabolic disease [14]. Moreover, early pubertal development is found to be associated with depression and eating disorders in children [15,16], while in adults it is associated with metabolic syndrome, type 2 diabetes, cardiovascular disease, breast cancer, and overall increased mortality [17,18,19,20]. Furthermore, exposure to early life adversity (e.g., family stressors, socioeconomic distress) resulted correlated with the timing of puberty [14,21].

In interpreting the phenomenon related to pubertal precociousness, an apparent incongruence seems to arise between the theories that explained the secular lowering of AAM, accompanied by accelerated growth, as due to improved environmental conditions (among others: [1]) and those that interpreted this decline, related to a high BMI and reduced stature growth, as the result of adverse living conditions [2]. In addition, the causal association of obesity with early menarche is still debated [22], partly because the studies that investigated the phenomenon were mostly cross-sectional [23]. In essence, therefore, the relationships between nutritional/weight status and early puberty are still little learned and their interpretation is not entirely clear.

The main purposes of this study are to estimate AAM in a sample of Italian girls aged 10–14 years, analyze whether an overweight or obesity condition results in earlier pubertal timing based on a longitudinal study, and, more generally, evaluate associations of menarche onset with growth and adiposity indices.

## 2. Materials and Methods 

### 2.1. Participants 

The current survey is part of a wider research designed to investigate the health and nutritional status, behaviors, and lifestyle of adolescent students in the Emilia-Romagna region (Italy). The research was approved by a local Bioethics Committee of the University (Bologna; Ethical Approval No. 2.18). 

In this longitudinal study, we specifically examined data on menarche onset in girls from a middle public school in the city of Ferrara. The participants were enrolled in a school chosen by a convenience criterion. All girls in the school were invited to participate through a school notice enclosing a copy of the informed consent to be signed by parents or guardians. Only girls who had obtained written consent before starting the survey and agreed to participate were included in the study. Twelve girls who did not have this consent were excluded. We planned to repeat the measures every six months starting in November 2019 in girls in the sixth, seventh and eighth grades (referred to in Italy as first, second, and third grade). However, it was not always possible to follow the organizational design of this longitudinal study due to the development in the same years of the COVID pandemic resulting in a ban on outside personnel entering schools. The study participants were 117 girls born between 2007 and 2010. No missing values were observed in survey questions or anthropometric measurements collected in the surveys conducted.

### 2.2. Assessment of Age at Menarche and Anthropometric Indices

Face-to-face interviews were carried out by the same operator (NR) to collect information on participants. Girls were asked the question if they had reached menarche. Respondents who gave affirmative answers were then requested to recall the precise date of their first menstruation (day, month, and year).

The same trained operator (LZ) performed all anthropometric measurements following standardized procedures [24,25,26,27]. Anthropometric measurements were taken in the morning on participants in light indoor clothing and without shoes. In all height measurements, participants were measured in a straightened position with their heads aligned in the Frankfurt plane. Standing height and sitting height were both measured to the nearest 0.1 cm by an anthropometer (Magnimeter, Raven Equipment Ltd., Dunmow, Essex, UK), while lower limb length was obtained as the difference between standing height and sitting height. Weight was determined to the nearest 0.5 kg with the use of a mechanical scale (SECA, Basel, Switzerland). Waist circumference was measured to the nearest 0.1 cm at an intermediate level between the lower margin of the lowest rib and the upper margin of the iliac crest using a non-stretchable metric tape. Skinfold thicknesses were taken at the triceps and subscapular points to the nearest 0.5 mm on the left side of the body according to Weiner and Lourie [25] using a Lange caliper (Beta Technology Inc., Houston, TX, USA). Further details on the anthropometric technique used have been reported previously [28].

Based on the above measurements, some anthropometric indices important for growth and body adiposity assessment were calculated. The skelic index was computed as (lower limb length/sitting height) ∗ 100. Body Mass Index (BMI) was computed as weight (kg)/height^2^ (m^2^). This index was used to classify participants in weight status categories (underweight, normal weight, overweight, obese) according to Cole cutoffs [29,30]. We computed the waist-to-height ratio (WHtR) as waist circumference divided by standing height. An increased health risk is defined for this index by values above the cut-off (0.50) [31,32]. The sum of triceps and subscapular skinfold thicknesses was used to estimate the percentage of body fat (%F) by Slaughter et al.’s equation [33] for girls.

Longitudinal data were used to calculate changes in anthropometric traits and indices and then turned into yearly growth rates (growth velocity, GV) by dividing the variation by the time elapsed over the two surveys.

### 2.3. Statistical Analysis

The Kolmogorov–Smirnov test was applied to check the normality of the variables. The values of triceps and subscapular skinfold thicknesses were log-transformed before comparisons.

We calculated percentage frequencies for qualitative variables (categories of BMI) and computed a chi-squared test to assess the difference between mature and non-mature girls by weight status categories. Kruskal–Wallis ANOVA was applied to test the difference among median values of AAM in the weight status categories.

Based on the last survey, means and standard deviations of anthropometric characteristics have been calculated separately for girls who had not yet had their first menstruation and girls who were already mature. As the two groups differed significantly in age, we applied ANCOVA adjusted for age to assess the differences.

Finally, within the subsample of mature girls (N = 54), we assessed the association (Pearson’s r) of the timing of puberty categories (by age at first menstruation: <11, 11, ≥12 years) with the anthropometric variables and their GV during the sixth grade. This was possible when two measurements spaced six months apart and the date of the AAM reached within the three-year period were available. The analyses conducted allowed us to analyze anthropometric GV at age 11 years at or before the changes in body fat that are part of the pubertal transition.

All statistical analyses were carried out using STATISTICA software, version 11 (StatSoft, Tulsa, OK, USA), and the significance level for all statistical tests was set at *p* < 0.05. 

## 3. Results

A total of 117 schoolgirls (age = 12.66 ± 0.98 years) were included in this longitudinal study setting. The median age of schoolgirls at the time of study entry was 11.34 years (range: 10.44–11.88 years).

Table 1 shows the mean values of anthropometric characteristics of the total sample at the last survey conducted in April 2022 and the cross-sectional statistical comparison based on the onset of menarche: 84 schoolgirls (71.8%) were already matured (mean chronological age = 12.92 ± 0.92 years) and the remaining 33 (28.2%) (mean chronological age = 12.02 ± 0.81) had not yet had menarche onset. 

The mean value of the skelic index falls into the macroskelic category, which means that the lower limb is long relative to the sitting height. The mean value of WHtR is lower than 0.5, and 16 schoolgirls (13.6%) exceeded the cut-off. The median AAM was 11.66 (95% IC: 11.31–11.68). The comparison between mature and non-mature girls, adjusted for age, highlighted mature girls had significantly higher mean values of sitting height and lower mean values of skelic index and WHtR than non-mature girls. 

Considering weight status in more detail (Table 2), the two groups of schoolgirls showed a significantly different distribution in the onset of menarche: normal weight was the most represented category in both, but underweight and obese categories were more represented in non-mature than in mature girls. Even if the AAM of girls in different weight status categories did not differ significantly by the Kruskal–Wallis ANOVA test, we remark that the mean value of AAM in the overweight category is the lowest.

Due to the longitudinal design of the study, we were able to share the sample in three classes of school girls according to the onset of the age at menarche (<11 years; 11 years ≤ age at menarche ≤ 12 years; >12 years). In order to consider possible associations with annual GV as well, we selected from the total sample a subsample of maturers (N = 54) who had two consecutive measurements in the first year of middle school.

Table 3 shows the correlation between anthropometric traits (absolute values in the first measurement, and their annual GV) and the category of menarche onset. The only variable that reached a value close to statistical significance was the skelic index with a positive coefficient: the school girls who mature after the age of twelve had higher values of skelic index.

## 4. Discussion

The numerous studies from the literature on AAM are generally based on self-reported data by women in adulthood, being then less reliable [34]. In addition, this type of retrospective study on adults undermines the possibility of verifying the anthropometric characteristics and growth rate during adolescence.

Given the supposed relationships between early pubertal time and health risk [14], we analyzed the age at the onset of menarche and growth characteristics of pre-pubertal/pubescent girls from an Italian middle school. The median AAM of 11.7 years obtained in this study is slightly lower than the mean value reported in a previous study carried out in the same region (Emilia-Romagna) (11.97 ± 0.94 years) [35] and for girls born approximately sixty years earlier (11.9 ± 1.4 years) always in the same region [10].

Furthermore, no association of BMI, weight, and adiposity indices with AAM was found in our study. This was evident both in comparisons between subsamples of mature and non-mature girls in cross-sectional analysis, and in the correlation analysis of anthropometric traits, adiposity indices, and relative GV with different age categories of pubertal development examined longitudinally. Further confirming this, the distribution of mature and non-mature girls in the different nutritional status categories (BMI) indicated no significant difference between categories. A correlation value close to statistical significance with the three categories of AAM resulted only for skelic index, consistently with the anthropometric changes in body proportion during pubertal growth.

Therefore, we discuss below our results concerning a moderate decline in AAM in the examined sample compared with results from an earlier study and the observed lack of association between AAM and adiposity indices, despite findings in some other studies.

Genetic and environmental factors can influence the physiological range of AAM. These factors have determined the well-known phenomenon of the secular trend at the population level.

The secular trend resulted in the first half of the 20th century in a significant decrease in AAM in Western countries. Italy also showed a clear decrease in mean AAM of 2 months each decade over a century by examining women born in the period from 1850–1859 to 1950–1959 and examined by a retrospective method in Bologna (Emilia-Romagna region) [10]. This trend, accompanied by an increase in standing height and other anthropometric changes in adults [36], has been interpreted as mainly related to an improvement in socioeconomic conditions, more adequate nutrition, and general health, similar to what has been argued for other European countries [6,37]. The median AAM obtained in this study is very close to the mean value of AAM computed for girls born approximately sixty years earlier (11.9 ± 1.4 years) confirming that the secular trend appears to have been halted in Italy around the 1960s as in other industrialized countries [6,38].

The pattern emerging from the comparison of the current AAM of Italian girls with those of girls of other European nations is consistent with the European picture characterized by a decreasing gradient of this variable from North to South Europe [7], confirming geographic diversities in growth and maturation that may be related to genetic or ethnic factors, as well as environmental factors [39].

In less advantaged populations, secular trends in AAM related to any changes in living conditions are still detectable. In this case, the effects of adverse socioeconomic conditions are particularly evident with delayed menarche and large variations in the timing and tempo of puberty between groups from different socioeconomic levels [40].

The above refers to detectable changes in physiological puberty at the population level. At an individual level, non-physiological female puberty (central precocious puberty, CPP) may result in the involvement of early pubertal changes (secondary sexual characteristics onset before the age of 8 years in girls), acceleration of growth velocity, and rapid bone maturation that often leads to a reduced adult height. This condition, sometimes brought about by unknown factors, first affects stage B2 with the onset of breast development (thelarche) at 7 years in Caucasian girls [41]. Precocious puberty has also been defined as the appearance of first signs of pubertal development at an age below 6.3 years in Caucasians, which statistically corresponds to 2 SD below the average [42]. However, age limits for sexual precocity are not definitively established [2]. Antoniazzi et al. [42] also consider it likely that the age limits between normal and precocious puberty should be lowered and it is uncertain whether the limits for the onset of puberty set in the US can also be considered valid for European populations. Clinically, CPP is distinguishable from normal puberty due to the presence of, but not limited to, signs of hypothalamic–pituitary–gonadal axis activation in girls under 8 years of age, bone age 2 SD higher than chronological age, increased growth velocity [42]. The mechanisms behind the increased incidence of this endocrine disorder are still uncertain, but several factors can affect the timing and tempo of puberty, such as genetics, epigenetics, lifestyle, nutrition, and exposure to the environment. Possible mechanisms involved in abnormal pubertal timing include prenatal and postnatal exposure to endocrine disruptors, as well as international adoptions [43]. In particular, early pubertal development has been related to exposure to certain categories of endocrine-disrupting chemical compounds such as pesticides, heavy metals, essential oils, etc. [44]. As for the increased risk of precocious puberty in immigrant and internationally adopted children, it is likely to depend on psychological stress and nutritional deprivation as well as on ethnic factors [4,45]. Among mechanisms that can induce early pubertal development, another important influence would be exerted by nutritional status (overweight/obesity) [3,43,46].

According to a recent study [7], 90% of Italian girls have the onset of menarche between the ages of 10 and 14 years. In our study, only 2 out of 117 girls had menarche at an age younger than 10 years (at 9.3 and 9.5 years). However, we have no indication of the age of onset of thelarche in these girls nor of other signs indicating nonphysiologic menarche.

It has been suggested that obesity may contribute to an early onset of AAM but the causal association of early menarche with obesity is still disputed in the literature [22]). While some studies claim that the age of pubertal onset is correlated with BMI [11,47], other studies found no correlation between these variables [48,49,50], as we confirmed in this study, or interpreted the increased adiposity as a consequence of sexual maturation in precocious girls [48,51]. According to studies conducted in the US by Lee et al. [52], obesity would simply favor an earlier onset of breast development but not the progression through the later stages of puberty. Therefore, the association between higher adiposity and pubertal acceleration does not necessarily provide evidence for a causal link but both traits could depend on faster neuroendocrine maturation [50,53].

Among the various reasons for these inconsistent findings in the studies of the literature, there are different methods to collect (status quo and recall) and report (mean or median values) the AAM data. Moreover, the age limits used to define early menarche vary depending on the research, even extending to include all those below the median age [54]. Finally, the cross-sectional design generally applied in these studies may have led to additional difficulties in interpreting the phenomenon.

In population studies, the claim of a decrease in AAM due to obesity should be considered with caution, always distinguishing such a trend from secular trends. In some cases (e.g., [55]), we believe that a different interpretation of the phenomenon is possible: the decline in AAM in girls of a developing country may be attributable to an ongoing secular trend phenomenon, and the observed correlations may be ascribed to two simultaneous events (menarche onset decline and BMI increase) that are non-causally interrelated.

Our study had several strengths and limitations. The major strengths of our study are the direct and rigorous measurement of anthropometric variables and its longitudinal design. A weakness of the current study is that we could not always conduct the six-monthly surveys in the school because of the COVID epidemic. In addition, our sample size is relatively small, and even more so are the subsamples based on weight status. Another limitation of this study is the use of retrospective data on menarche onset. Anyway, despite different findings in the accuracy of AAM referred by women in adulthood [34,56], AAM reported by schoolgirls at a short distance from the event (days or months) is believed to be more reliable [3]. Indeed, we assessed pubertal timing by real-time self-reports of AAM during repeated surveys every six months. There is also a lack of socioeconomic information on the girls’ families, which is an additional limitation, given the influence that socioeconomic status can have on pubertal development.

## 5. Conclusions

With this study, we confirmed that the secular trend no longer resulted in any decrease in AAM for Italian girls, who show similar values as women born 60–70 years ago in the same region. In addition, the associations between menarche onset and adiposity indices are non-significant in the sample examined, involving moderate/no influence of the latter on the AAM for the age range considered. Future research is expected to confirm these findings, establish the limits of physiological puberty taking into account ethnic variability, and examine what factors may favor or exclude associations between AAM and body composition. We suggest that female puberty be analyzed longitudinally as early as elementary school to monitor pubertal development and capture all possible signs of accelerated growth and anthropometric changes until the onset of first menstruation. 

## Figures and Tables

**Table 1:** Anthropometric characteristics of the sample by the onset of menarche.

	Total Sample (N = 117)		Mature (N = 84)		Non-Mature (N = 33)		ANCOVA	
	Mean	SD	Mean	SD	Mean	SD	F	p
Standing height (cm)	156.1	6.9	157.3	6.5	152.8	6.9	2.793	0.097
Weight (kg)	52.0	12.1	52.6	10.2	50.4	16.3	0.047	0.828
Sitting height (cm)	81.9	3.7	82.8	3.1	79.7	4.1	7.492	**0.007**
Lower limb length (cm)	74.1	4.4	74.5	4.3	73.2	4.6	0.066	0.799
Waist circumference (cm)	68.1	9.3	67.5	7.3	69.7	13.1	2.248	0.137
Triceps skinfold (mm)	16.7	6.0	16.8	5.4	16.5	7.4	0.085	0.771
Subscapular skinfold (mm)	13.2	6.9	12.9	5.9	14.0	9.1	0.117	0.733
%F	24.9	7.7	24.9	6.6	25.0	10.0	0.403	0.527
BMI (kg/m^2^)	21.3	4.3	21.2	3.5	21.4	5.9	0.581	0.447
Skelic index	90.5	5.1	90.0	4.4	92.1	6.3	3.721	**0.042**
WHtR	0.44	0.06	0.43	0.04	0.45	0.08	3.520	**0.049**

Note: comparisons for skinfold thicknesses were performed using log skinfolds.

**Table 2:** Distribution and age at menarche (AAM) of the sample by weight status categories.

	Total Sample (N = 117)	Mature (N = 84)	Non-Mature (N = 33)		AAM (N = 84)		
Weight Status	N (%)	N (%)	N (%)	p	mean	SD	p
Underweight	5 (4.3)	1 (1.2)	4 (12.1)	**0.024** ^§^	11.60	-	0.2731 *
Normal weight	69 (59.0)	53 (63.1)	16 (48.5)		11.80	0.88	
Overweight	35 (29.9)	26 (31.0)	9 (27.3)		11.34	0.77	
Obese	8 (6.8)	4(4.8)	4 (12.1)		11.56	1.15	

Note: ^§^ calculated by chi-squared test; * calculated by Kruskal–Wallis ANOVA test.

**Table 3:** Pearson correlation between anthropometric variables and categories of AAM (<11 years; 11 years ≤ age at menarche ≤ 12 years; >12 years) (N = 54).

Anthropometric Traits	r	r^2^	t	p
Standing height	0.071	0.005	0.502	0.618
Weight	−0.139	0.019	−0.992	0.326
Sitting height	−0.054	0.003	−0.379	0.706
Lower limb length	0.150	0.022	1.072	0.289
Waist circumference	−0.144	0.021	−1.032	0.307
Triceps skinfold	0.008	0.000	0.059	0.953
Subscapular skinfold	−0.122	0.015	−0.870	0.388
%F	−0.055	0.003	−0.391	0.698
BMI	−0.214	0.046	−1.553	0.127
Skelic index	0.262	0.068	1.954	0.056
WHtR	−0.164	0.027	−1.174	0.246
**Annual Growth Velocity**				
Standing height	0.187	0.035	1.344	0.185
Weight	0.148	0.022	1.059	0.295
Sitting height	0.187	0.035	1.343	0.185
Lower limb length	0.056	0.003	0.400	0.691
Waist circumference	0.250	0.063	1.828	0.074
Triceps skinfold	−0.114	0.013	−0.831	0.410
Subscapular skinfold	−0.025	0.001	−0.183	0.855
%F	−0.093	0.009	−0.657	0.514
BMI	0.109	0.012	0.774	0.443
Skelic index	−0.048	0.002	−0.342	0.734
WHtR	0.224	0.050	1.628	0.110

## Data Availability

Data are available upon request due to ethical restrictions regarding participant privacy. Requests for the data may be sent to the corresponding authors.
